# Supplementary material for: Causal interplay between lactose intolerance and gut microbiota: a combined bidirectional Mendelian randomization and in vivo validation study
Source: Front Nutr. 2026 Jun 1;13:1803337. doi: 10.3389/fnut.2026.1803337 (PMC13265576; doi:10.3389/fnut.2026.1803337)
Supplement: Supplementary file 1 [file Data_Sheet_1.zip › supplementary materials/Forward/forest plot/ebi-a-GCST90027708.finngen_R12_E4_LACTONAS.pdf]

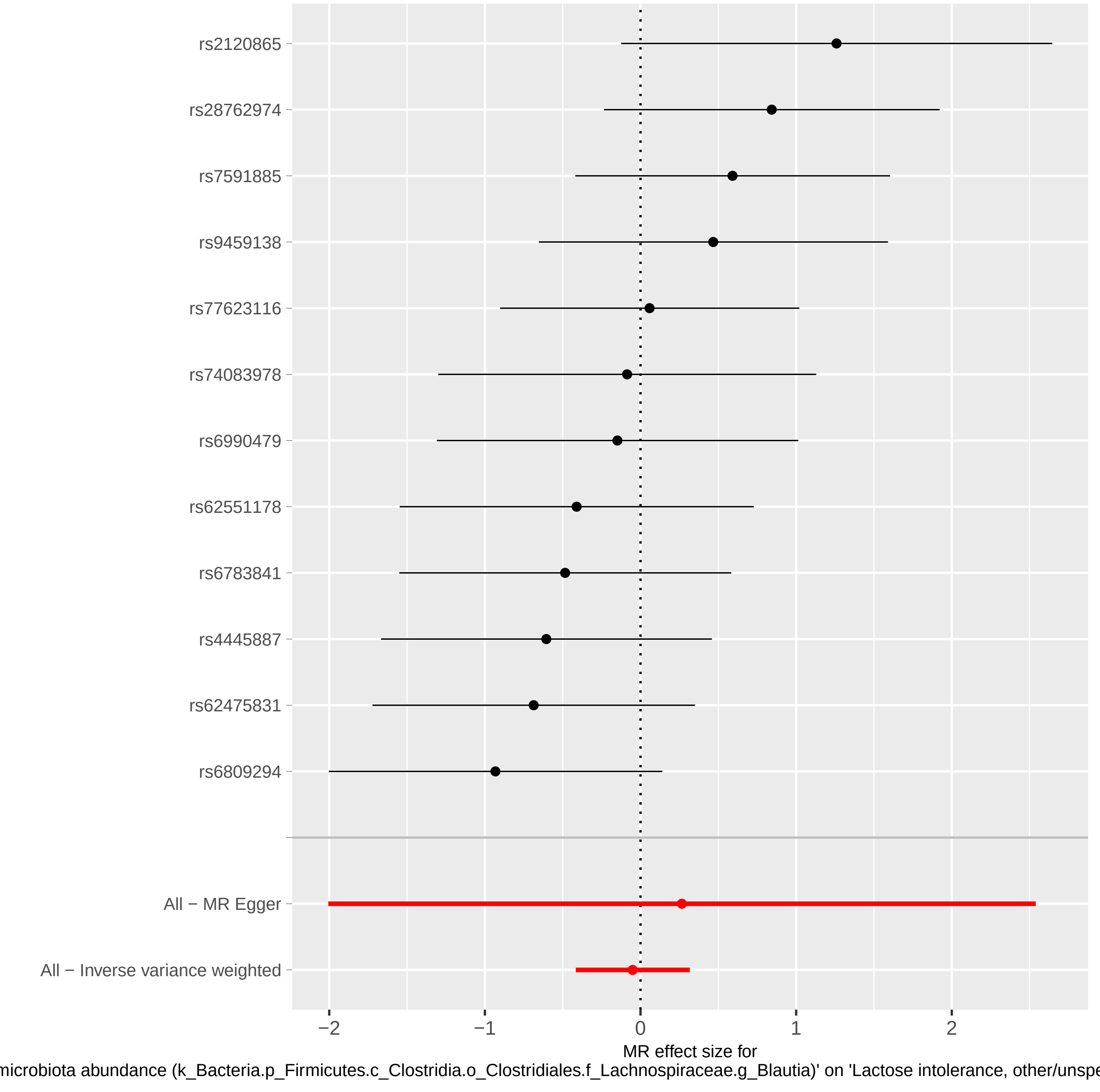

rs2120865
rs28762974
rs7591885
rs9459138
rs77623116
rs74083978
rs6990479
rs62551178
rs6783841
rs4445887
rs62475831
rs6809294
All − MR Egger
All − Inverse variance weighted
−2
−1
0
1
2
MR effect size for
'Gut microbiota abundance (k_Bacteria.p_Firmicutes.c_Clostridia.o_Clostridiales.f_Lachnospiraceae.g_Blautia)' on 'Lactose intolerance, other/unspe
